# New insights for an old drug: difluoromethylornithine boosts the trypanocidal action of benznidazole on *Trypanosoma cruzi in vitro* and *in vivo*

**DOI:** 10.3389/fcimb.2026.1794141

**Published:** 2026-03-30

**Authors:** Santiago José Martínez, María Cristina Vanrell, Xiaomo Li, Carolina Carrillo, David M. Engman, Patricia Silvia Romano

**Affiliations:** 1 Laboratorio de Biología de Trypanosoma cruzi y la célula hospedadora - Instituto de Histología y Embriología “Dr. Mario H. Burgos”, IHEM-CONICET- Universidad Nacional de Cuyo, Mendoza, Argentina; 2 Cedars-Sinai Medical Center, Los Angeles, CA, United States; 3 Área Biología Celular y Molecular, Facultad de Ciencias Médicas, Universidad Nacional de Cuyo, Mendoza, Argentina; 4 Instituto de Ciencia y Tecnología Dr. César Milstein, ICT Milstein-CONICET, Buenos Aires, Argentina

**Keywords:** benznidazole, Chagas disease, combined therapies, difluoromethylornithine, *Trypanosoma cruzi*

## Abstract

Chagas disease, caused by the protozoan parasite *Trypanosoma cruzi*, is currently a global health problem. Its multiple forms of transmission, which favor the spread of the infection, and the limited treatment options—still restricted to only two drugs—highlight the urgent need for research and development in the field. Despite certain disadvantages, the potent trypanocidal activity of benznidazole (BNZ) and nifurtimox explains their continued use notwithstanding their high toxicity. Reducing BNZ doses through combination with another antiparasitic drug is an attractive strategy to improve tolerability while maintaining efficacy. In this work, we explored the anti-*T. cruzi* effect of BNZ in combination with difluoromethylornithine (DFMO), a repurposed drug currently used for the treatment of certain cancers and other pathologies. Our data showed that DFMO increases parasite susceptibility to BNZ, reducing the IC50 by half compared to BNZ monotherapy *in vitro* across strains from three different *T. cruzi* genetic lineages. Subsequently, we examined the effect of the combination *in vivo*. We found that mice treated with a 10-fold lower dose of BNZ combined with DFMO displayed a lower parasitemia peak and reduced tissue parasitosis during the acute stage, as well as less parasite reactivation in organs after immunosuppression in the chronic stage, compared to BNZ monotherapy. Overall, these findings support the BNZ/DFMO combination as a proof of concept for Chagas disease therapy, offering a viable strategy to mitigate BNZ-related toxicity through dose reduction without compromising therapeutic efficacy.

## Introduction

*Trypanosoma cruzi* (*T. cruzi*) is the etiologic agent of Chagas disease, a vector-borne and life-threatening illness endemic in 21 countries of Latin America, where the insect vectors are found. Chagas disease is also prevalent in non-endemic countries due to migration flows and the risk of vertical transmission, which currently represents the most significant route of infection in these regions (Antinori et al., 2017). Blood transfusion, organ transplant, laboratory accidents, or ingestion of food or drinks contaminated with parasites complete the list of infection modes and depict the high versatility of *T. cruzi* to gain access to the host by different routes (Chagas disease, WHO, 2021). Based on its genetic diversity, *T. cruzi* is classified into seven lineages or Discrete Typing Units (DTUs), from TcI to TcVI and the bat-associated form, TcBat (Lima et al., 2015; Zingales and Macedo, 2023). Human infections in domestic transmission cycles are primarily caused by strains from TcI, TcII, TcV, and TcVI, whereas TcIII and TcIV are typically linked to the sylvatic cycle and oral transmission outbreaks (Zingales and Macedo, 2023).

Chagas disease progresses through two successive stages: the acute phase, with patent parasitemia, and the chronic phase, characterized by sub-patent parasitemia and scarce tissue parasitism. The majority of chronically-infected individuals are asymptomatic, whereas up to 30-40% of patients develop chronic cardiomyopathy or gastrointestinal disorders. While Chagas disease pathology is poorly understood, accumulating evidence indicates that parasite persistence within the target organs, in concert with an unbalanced immune response, plays a central role in the development of pathology in both the acute and chronic phases. In agreement with this concept, it is recommended that Chagas disease be treated with anti-parasitic compounds regardless of the stage of *T. cruzi* infection (Dias Gontijo and Galvão, 1999).

Benznidazole (BNZ) and Nifurtimox (NFX) are the only two drugs approved for Chagas disease treatment to date. They are nitroheterocyclic prodrugs which share a nitro group essential for activation by parasitic nitroreductases (TcNTRs). BNZ generates cytotoxic radicals that alkylate DNA, proteins and lipids, while NFX induces oxidative damage and lipid peroxidation, both impairing cell replication and growth and leading to cell death. Although with a high profile of parasitological cure in the acute infection, efficacy of these drugs is progressively reduced throughout the chronic phase. Therapeutic failure is also associated with the required high dose for a long time (60-day regimen), and the frequent occurrence of serious side effects which reduce patient adherence.

Drug resistance is another key factor in the treatment efficacy. Resistance to BNZ is a complex phenomenon that occurs naturally among *T.cruzi* strains. Enhanced antioxidant defenses (e.g., superoxide dismutase), alterations in DNA repair pathways, and mutations in the TcNTR gene that impair BNZ activation have all been associated with reduced drug susceptibility (Campos et al., 2014; MaChado-Silva et al., 2016). Using a BNZ naturally-sensible and resistant strains (from DTU TcI), Ospina and colleagues found that trypanothione synthase and thioredoxin are overexpressed in the BNZ resistant DA strain (Ospina et al., 2025). Trypanothione or bis-glutationyl spermidine (T(SH)_2_) is a central molecule of trypanosomatids that neutralize oxygen radicals and protect against parasitic oxidative stress induced by BNZ.

Spermidine (Spd), the precursor of T(SH)_2_, belongs to the group of polyamines (PAs), essential compounds for eukaryotic organisms. PAs are basic molecules necessary for DNA and RNA stability, and the correct function of other biomolecules, proteins and lipids. They regulate fundamental cellular processes, cell growth, proliferation and differentiation (Pegg, 2016). The biosynthesis of PAs begins with the oxidative decarboxylation of ornithine, catalyzed by the enzyme ornithine decarboxylase (ODC), to yield putrescine (Put). Subsequently, Put is converted into Spd and spermine (Spm) through the sequential action of spermidine and spermine synthases. In contrast to *Trypanosoma brucei* and other trypanosomatids, *T. cruzi* lacks ODC, the rate-limiting enzyme in PA biosynthesis (Carrillo et al., 1999). To compensate for this auxotrophy, *T. cruzi* acquires PAs from the extracellular medium through specialized transporters, such as TcPAT12 (Carrillo et al., 2006).

The ornithine analog DL-α-difluoromethylornithine (DFMO, eflornithine) was initially developed as an anticancer agent due to its irreversible inhibition of ODC, which triggers cell cycle arrest by depleting intracellular PA levels (Bassiri et al., 2015). Eflornithine was subsequently found to be effective against Human African Trypanosomiasis (HAT), offering a safer alternative to arsenic-based chemotherapy (Docampo and Moreno, 2003). We previously demonstrated that mammalian cells incubated with 1 mM DFMO for 48–72 h exhibit significantly reduced intracellular spermidine (Spd) levels without compromising cell viability (Vanrell et al., 2013a). Given the dependence of *T. cruzi* on host Spd for trypanothione production, we investigated whether DFMO could enhance the parasite’s susceptibility to benznidazole (BNZ). We hypothesized that DFMO-mediated reduction of host Spd levels impairs trypanothione synthesis in the parasite, thereby increasing its sensitivity to the cytotoxic molecules generated by BNZ and NTX. This study explores the repurposing of DFMO as an adjuvant to enhance BNZ efficacy *in vitro* and *in vivo*, aiming to maintain therapeutic effectiveness while minimizing dose-dependent toxicity.

## Methods

### Drugs and reagents

Dulbecco-modified minimal essential medium (DMEM - high glucose) and the fetal bovine serum (FBS) were purchased from Serendipia Lab. (Buenos Aires, Argentina). The rabbit anti-*T. cruzi* polyclonal antibody was kindly provided by Dr. Catalina Alba-Soto (Instituto de Investigaciones en Microbiologia y Parasitologia Médica (IMPAM-UBA-CONICET, Buenos Aires, Argentina) and the immune serum against *T. cruzi* raised in C57BL/6 mice was developed in our laboratory. The anti-mouse Cy3, and the anti-rabbit Cy3 secondary antibodies were purchased from Jackson ImmunoResearch Laboratories (West Grove, PA, USA) and from Life Technologies (Buenos Aires, Argentina), respectively. The pTRIX-mNeonGreen-LUC plasmid was generously gifted by Dr. John M. Kelly (Department of Pathogen Molecular Biology, London School of Hygiene and Tropical Medicine, London, UK). The other reagents, L-glutamine, penicillin, streptomycin, and the rhodamine-conjugated phalloidin were obtained from Invitrogen (Buenos Aires, Argentina). The alamarBlue reactive was purchased from Biosource-Life Technologies, the Hoechst 33342 dye from Life Technologies (Buenos Aires, Argentina), and the Mowiol 4–88 from Calbiochem, Merck (Buenos Aires, Argentina).

Benznidazole (BNZ), DL-α-difluoromethylornithine (DFMO), D-luciferin, and cyclophosphamide were purchased from Sigma-Aldrich (St. Louis, MO, USA). Stock solutions (100 mM BNZ and 1 M DFMO) were prepared in dimethyl sulfoxide (DMSO) and stored at -20 °C. In all experiments, the final concentration of DMSO did not exceed 0.1% (v/v) to avoid cytotoxic effects.

### Growth and maintenance of cell lines

Vero C-76 cells (ATCC^®^ TL-1456) (African green monkey kidney cells) and H9c2 (rat embryonic cardiomyocyte cells) (ATCC^®^ CRL1446 ™) were cultured in D-MEM supplemented with 10% of FBS, 100 units/mL penicillin, and 100 µg/mL streptomycin in a controlled atmosphere (5% CO2, 80% humidity, 37 °C). Adherent cell lines were detached with 0.05% trypsin-0.02% EDTA solution when 90% of confluence was reached. The cell suspension was pelleted by centrifugation, counted in a Neubauer chamber and subsequently prepared for cell infection experiments.

### *T. cruzi* transfection

Bioluminescent *T. cruzi* Tulahuen strain (Tul-Luc-mNeonGreen) was generated by transfection of epimastigotes with the Luc-mNeonGreen plasmid following the protocol described by Martin C. Taylor and colleagues (Taylor et al., 2019). Briefly, a total of 1 x 10^8^ epimastigotes were transfected by electroporation with the dual reporter plasmid to express a fusion protein comprising red-shifted luciferase and green fluorescent protein domains (pLuc-mNeonGreen). The parasites that successfully integrated the plasmid were monitored by fluorescence microscopy. Transfected epimastigotes were selected and maintained in 100 μg/ml geneticin (G418, Thermo Scientific™) as previously described (Costa et al., 2018). Bioluminescent trypomastigotes generated through spontaneous differentiation were recovered from the cultures of transfected epimastigotes by infection of H9c2 cells following previously established laboratory protocols (Barclay et al., 2011).

### Proliferation and enrichment of trypomastigotes

Tissue-cell derived trypomastigotes (TCT) were obtained from cell culture as follows. H9c2 or Vero cells at 30% of confluence were infected with trypomastigotes of Tul-Luc-mNeonGreen, Y-GFP or TcM strains. These cultures were incubated for 3 days in D-MEM supplemented with 3% FBS, followed by washing and replacement with fresh medium to eliminate extracellular parasites. After 4 to 6 days, highly motile intracellular trypomastigotes caused cell lysis and reached the medium. Free trypomastigotes were harvested in a 15 ml tube and centrifuged at 2000 g for 10 minutes. The pellets were incubated with fresh medium at 37 °C and 5% CO_2_ for 3 h to allow the swim up of motile trypomastigotes into the supernatant. The trypomastigote enriched supernatant was collected, counted in the Neubauer chamber and used to infect cells.

### Cell viability test

20,000 H9c2 embryonic cardiomyocyte cells were cultured at 37 °C and 5% CO_2_ in DMEM supplemented with 10% FBS, Penicillin + Streptomycin in a 24-well plate, in the presence of BNZ (from 0 to 250 mM) and DFMO (from 0 to 5 mM) in a checkerboard dose-matrix. After 48 h treatment, AlamarBlue™ indicator dye was added to each well and incubated by an additional period of 6 h following the protocol previously described (Vanrell et al., 2013a). The resulting supernatants of each condition were measured in a 96-well plate using the colorimetric reader Lumistar Omega BMG LABTECH at 540 nm.

### Cell infection assays

H9c2 cells were plated in 8-well cell culture slides (75000 cells in a volume of 3 ml, Biologix^®^), or 24-well plates on round-shaped glass coverslips (25000 H9c2 cells in a volume of 1 ml); and infected with TCT of Tul-Luc-mNeonGreen, Y-GFP or TcM strains for 24 h using a MOI (Multiplicity of infection) of 10. Plates were then washed 3 times with 1X PBS to remove the trypomastigotes that had not entered the cells and incubated for an additional period of 48 h in the presence of 1 mM DFMO and increasing concentrations of BNZ (from 0 to 100 μM) both dissolved in DMSO as vehicle. Cells were then washed, fixed and prepared for microscopy analysis. The intracellular parasites (amastigotes) were directly detected by the green fluorescence of transfected parasites or by indirect immunofluorescence using specific anti-*T. cruzi* antibodies followed by a secondary antibody fused with a fluorescent marker. The host cell cytoplasm was stained with rhodamine-phalloidin that bound to the F-actin as described below.

### Indirect immunofluorescence assays

For the **i**ndirect immunofluorescence assay (IFA), cells were fixed with 4% PFA (paraformaldehyde) in PBS for 30 minutes at Room Temperature (RT), washed three times with 1X PBS, incubated with 50 mM NH_4_Cl for 15 minutes to quench the remaining free aldehyde groups, and then incubated with the permeabilization solution (1% Albumin/0.05% Saponin in PBS) for 20 minutes at RT. Samples were then incubated with the primary polyclonal anti-*T. cruzi* serum generated in mice (1:100), ON at 4° C. After washing in 1x PBS, cells were incubated with anti-mouse Alexa Fluor^®^ 488 (1: 100) for 2 h at 37 °C. F-actin of host cells were then stained by incubation with rhodamine-phalloidin reactive (1: 200) for 1 h at 37 °C. The slides were finally mounted with Mowiol containing Hoechst (1: 1000) for DNA staining. Fluorescent images were acquired in a Revolve 4 Upright- Inverted-Fluorescence Microscope, Echo Laboratories (San Diego, CA, USA) or in a Nikon Upright 80i Eclipse fluorescence microscope (Nikon). Image processing was done with Image J version 1.53 m (Fiji).

### Animal and ethics statements

All animal protocols were reviewed and approved by the Institutional Animal Care and Use Committee of Cedars-Sinai Medical Center, Los Angeles, California, USA. (ACUC007053). Animals were purchased from Jackson Laboratory and were maintained under pathogen free conditions and 12-h dark/light cycle at a temperature 22 + 3 °C. They have access to food and water *ad líbitum*. Female mice aged 8–12 weeks were used in all experiments. For bioluminescence imaging, animals were anesthetized via inhalation of 3% (vol/vol) isoflurane in oxygen. At the conclusion of the study, or upon reaching a predefined humane endpoint, mice were euthanized by cervical dislocation while maintained under a deep plane of anesthesia.

### *T. cruzi* preparation for mice infection

Parasites for mice experiments were obtained from the blood of SCID (Severe Combined Immunodeficiency) mice infected with trypomastigotes of bioluminescent *T. cruzi* Tulahuen strain (Luc-mNeonGreen) collected from supernatants of H9c2 cells. Infection was performed intraperitoneally (IP) with 5000 trypomastigotes. The bioluminescence intensity was monitored every 3–5 days by analysis of luciferase activity as described previously (Taylor et al., 2021). Briefly, after IP injection of 150 mg/kg d-luciferin (GOLDBIO^®^), animals were anaesthetized using 3% (vol/vol) gaseous isoflurane/oxygen and placed in an IVIS Spectrum (Caliper Life Science) equipment. Bioluminescence intensity was then acquired and analyzed with the Softmas Pro Software with an exposure time between 5 to 60 s, depending on signal intensity. At high intensity signal, when parasitemia reached 1x10^8^ per ml, blood was collected from the saphenous vein and parasites were prepared for animal experiments.

### Animal model and treatment protocol

Mice infections were performed with trypomastigotes from the bioluminescent *T. cruzi* Tulahuen strain obtained from the blood of SCID mice. Healthy adults (8-12-week age) female BALB/c mice were infected with 5000 trypomastigotes by IP injection. Drug treatment started at day 7 post-infection (7 DPI), and extended 2 weeks up to 21 DPI. Mice were treated by oral gavage doses of 100 mg/kg/day or 10 mg/kg/day BNZ dissolved in a 2% methyl-cellulose solution with sterilized water and administered with disposable sterile flexible Teflon gavage needles. DFMO (200 mg/kg every 2 days) was diluted in sterile physiologic solution and inoculated by IP injections. The bioluminescence intensity was measured as described above and determined every 3, 4 or 7 days until the end of the experiment. At the 42 DPI, a 2-week immunosuppression treatment was done by IP injections of 200 mg/kg cyclophosphamide dissolved in sterilized water and administered every 3 days. At the end of the treatment, mice were euthanized via cervical dislocation under deep anesthesia (3% vol/vol gaseous isoflurane/oxygen) and the organs of one mouse randomly selected were harvested and injected with d-luciferin following the method described at (Lewis et al., 2015).

### Statistical evaluation

All statistical analyses were performed with GraphPad Prism version 8.0.0 for Windows (GraphPad Software, San Diego, California USA, www.graphpad.com). The data were represented as the mean and standard error of the mean (SEM). Student’s t-test was used for straightforward pair-wise comparisons between the two groups. ANOVA was employed for multiple comparisons, followed by the Bonferroni correction. Statistical significance was defined as a p-value less than 0.05.

## Results

### DFMO increases the susceptibility of *T. cruz*i to BNZ treatment *in vitro*

To evaluate the effect of the drugs on culture cells, we first assessed the cytotoxicity of BNZ and DFMO (both individually and in combination) on H9c2 rat embryonic cardiomyocytes. Cell viability was measured using the Alamar Blue assay after incubating the cells with increasing concentrations of both compounds in a checkerboard dose-matrix. The results indicated that the combination of 100 µM BNZ and 1 mM DFMO was the highest concentration of each drug that maintained cell viability near 100% (**Supplementary Figure 1**). Since 1 mM DFMO has been shown to significantly reduce host cell Spd (Vanrell et al., 2013a), we set the treatment parameters for H9c2 cells in dose-response assays at 0.1–100 µM BNZ in the presence of a constant concentration of 1 mM DFMO.

**Figure 1:**
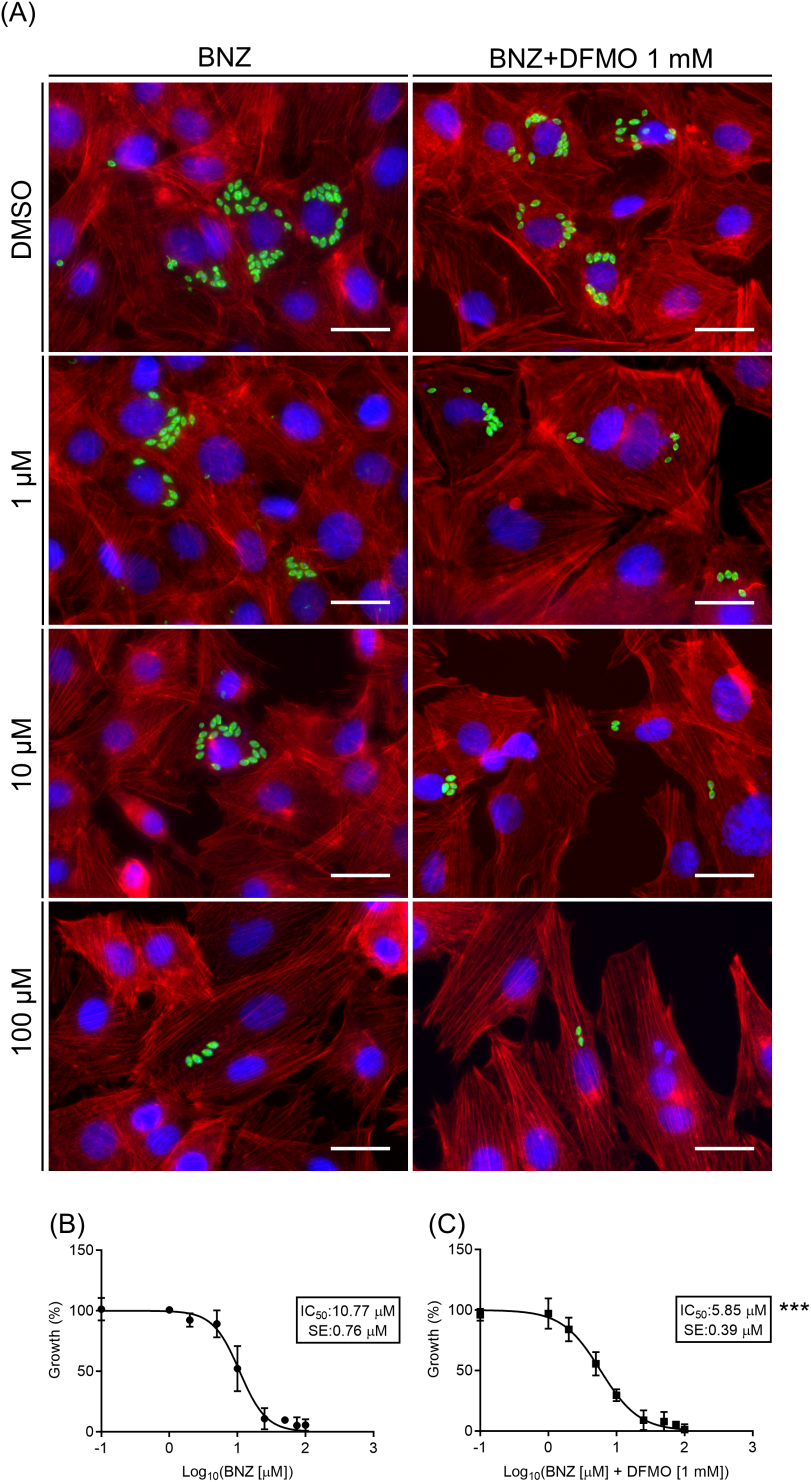
Antiparasitic effect of benznidazole monotherapy and the combination BNZ/difluoromethylornithine *in vitro*. H9c2 cells were infected with trypomastigotes of *T. cruzi* Tul-Luc-mNeonGreen strain (MOI = 10) for 24 h followed by a chase of 48 h in the presence of the indicated concentrations of benznidazole (BNZ) either alone or in combination with 1 mM DFMO (BNZ + DFMO). Controls were treated with the vehicle, DMSO. After fixation, cells were prepared for microscopic studies. **(A)** Representative confocal images depicting amastigotes detected by IFA (green) within the host cells at 48 h of incubation with the indicated treatments. H9c2 cells actin myofibrils were stained with the rhodamine-phalloidine probe (red) and the host and parasite DNA were labeled with Hoechst 33342 (blue). **(B)** Dose-response curves of BNZ and BNZ + DFMO **(C)** against the % of amastigote growth in each concentration. Data points represent the mean +/- SE of three independent experiments performed in triplicate. The IC_50_ values were calculated using non-linear regression (sigmoidal dose-response variable slope). Significance levels established by p-values **p ≤ 0.01 compared to BNZ monotherapy.

Infection experiments were subsequently conducted to evaluate the effect of the BNZ/DFMO combination on *T. cruzi* intracellular growth (see details in methods). As shown in **Figure 1A**, the number of amastigotes decreased as BNZ concentration increased in the medium (left panels). The addition of DFMO further enhanced this reduction at every BNZ concentration tested, resulting in significantly lower parasite counts compared to those observed with BNZ alone (right panels). Importantly, host cell numbers remained unaffected by the treatments, which is consistent with our cytotoxicity assay results. Dose-response curves for each condition are depicted in **Figures 1B, C**, respectively. The IC50 calculated for BNZ at these conditions was 10.77 +/- 0.76 μM, whereas it was 5.85+/- 0.39 μM when combined with DFMO. Similar experiments were performed with two additional *T. cruzi* strains: the Y-GFP strain (DTU-TcII) and TcM (DTU-TcV). The latter is a clinical isolate obtained from a patient with chronic Chagas disease who experienced acute reactivation following immunosuppressive therapy (Martinez et al., 2022). In all cases, treatment with BNZ in the presence of DFMO reduced the IC50 by around 40 - 50% in comparison with BNZ monotherapy (**Table 1**).

**Table 1:** IC_50_ values calculated from infection assays performed as explained in **Figure 1**, using H9c2 cells and trypomastigotes of *T. cruzi* from different genetic lineages, Tul-Luc-mNeonGreen (DTU VI), Y-GFP (DTU II), and TcM (DTU V).

Strain	IC50 BNZ (μM)	IC50 BNZ/DFMO (μM)	% reduction
Tul-Luc-mNeonGreen	10.77 +/- 0.76	5.85 +/- 0.39	46
Y-GFP	21.17 +/- 1.14	12.39 +/- 1.83	41.5
TcM	15.91 +/- 1.90	7.75 +/- 1.25	51.3

The percentage of reduction is also indicated (see details in methods).

### BNZ/DFMO combination improves the infection control *in vivo*

**Figure 2:**
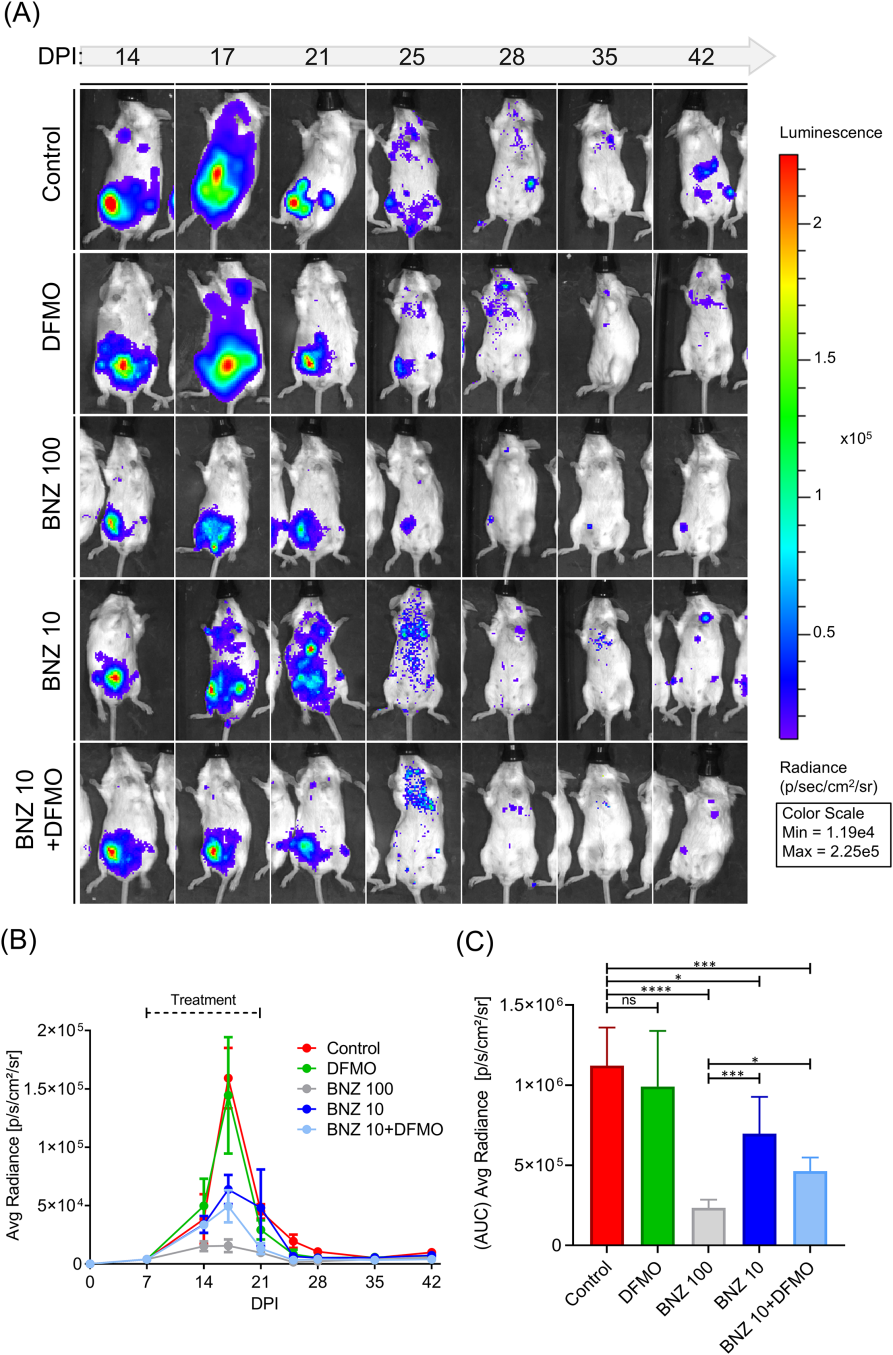
Live imaging of bioluminescent parasites on infected mice during treatment. Five groups of 5 mice each were infected with 5000 trypomastigotes of Tulahuen Luc-mNeonGreen double mutant strain. At 7 DPI, animals from each group were treated with 10 or 100 mg/Kg/day BNZ (BNZ 10 and BNZ 100, respectively), 200 mg/kg DFMO every 2 days (DFMO) and 10 mg/Kg/day BNZ plus 200 mg/kg DFMO every 2 days (BNZ + DFMO) for a period of 14 days and periodically measure their body luminescence (see details methods). Control animals were treated with the vehicle, DMSO. **(A)** Bioluminescence imaging of representative infected mice showing the level of parasitism of each group. The right panel indicates the luminescence scale. **(B)** The scatter curve graph shows the mean ± SD of radiance measurements obtained from each group at different days after infection. **(C)** Bars showed the mean and the SE of the area under the curve obtained from each group from 7 to 42 DPI. One-way ANOVA statistical analysis was performed with Dunnet multiple comparison tests. Significance levels established by p-values *≤0,05; ***≤0,001; ****≤0,0001.

The effect of BNZ/DFMO combination was next analyzed *in vivo* on a mouse model of *T. cruzi infection*. BALB/c mice were infected with a bioluminescent *T. cruzi* strain expressing the dual reporter luciferase and NeonGreen genes (*T. cruzi* LucNeonGreen) which allowed studying the curse of infection by the luminescence intensity emitted by parasites after injection of luciferin (see details in methods). Mice were divided in 5 groups of 5 mice each according to the treatments. Drugs vehicles were administered in the control group and the rest of groups were treated with DFMO (200 mg/kg every 2 days), BNZ high and low dose (100 and 10 mg/Kg/day, respectively), and the combination BNZ low (10 mg/Kg/day) plus DFMO. Luminescence detection was performed at 3, 4 or 7 days post-infection (DPI) to follow the infection in each group. Images in **Figure 2A** showed the location and distribution of *T. cruzi* throughout the body of one representative mouse of each group. The colorimetric scale in the right panel shows the colors spectrum related to the magnitude of the infection, with red being the maximum and violet the minimum radiance. High infection levels were evident in the peritoneal area, where *T. cruzi* was injected. Infection was detected at 14 DPI, spreading to various organs and tissues before decreasing around 25–28 DPI, with residual signals persisting thereafter. The average radiance in each group is plotted in the graphic of **Figure 2B**. The luminescence signal increased from 7 to 14 DPI, had a peak at 17 DPI, and next decreased at 21–28 DPI. Low signals were detected after that (35 and 42 DPI) in agreement with the progression to the chronic infection stage where the levels of parasitemia are reduced to values ​​close to 0 but the parasites are still present in certain tissues. All groups displayed similar curves with a strong reduction of the peak in the BNZ 100 group (gray curve) followed by BNZ 10/DFMO and BNZ 10 groups. Mice treated with DFMO displayed a curve very similar to the control group, indicative of no effect of this individual drug on the course of infection.

To analyze the overall efficacy of the different treatments, we calculated the Area Under the Curve (AUC) for each group and compared them using a bar chart (**Figure 2C**). As expected, no significant differences were found between the control and DFMO-only groups. The BNZ 100 group showed the greatest reduction, with very low AUC value (P<0.0001). Notably, the low-dose groups (BNZ 10 and BNZ 10/DFMO) also showed significant decreases in AUC values. However, the combined treatment (BNZ 10/DFMO) achieved a higher level of significance (*P* = 0.0005) than the BNZ 10 monotherapy (*P* = 0.0243) relative to the control. Further analysis between the BNZ groups displayed that, while the BNZ 10/DFMO group was nearly as effective as the BNZ 100 group (showing only a marginal difference; *P* = 0.0495), the BNZ 10 monotherapy remained strikingly different from the high-dose BNZ 100 (*P* = 0.0005). These data confirm the synergistic effects of the drug combination *in vivo*, suggesting that DFMO enhances the efficacy of BNZ treatment at low doses.

**Figure 3:**
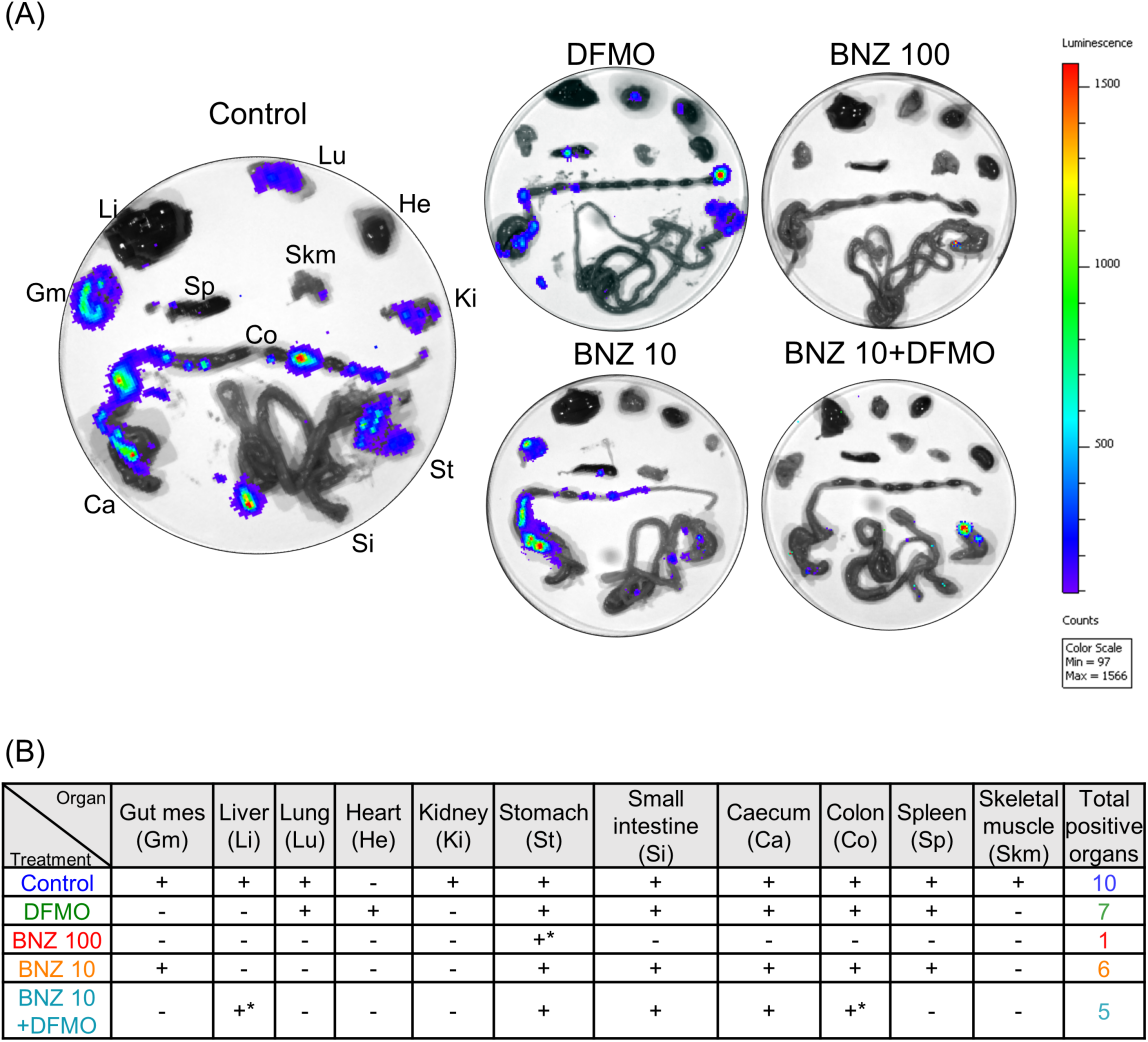
Infection recoveries after immunosuppression in the chronic stage. At 42 DPI animals were injected with 200 mg/kg cyclophosphamide during 2 weeks. After luciferin injection animals were euthanized for the post mortem imaging of organs. **(A)** The image in the left panel indicates the arrangement and the luminescence of organs and tissues from control animals (treated with the vehicle DMSO). Other images show the luminescence of organs from the animals at the indicated treatments. **(B)** The table summarizes the presence (+) or absence **(-)** of *T. cruzi* reactivation in the organs and tissues obtained from animals under each treatment. GM, Gut mes; Li, liver; Lu, lung; He, heart; Ki, kidney; St, stomach; Si, small intestine; Ca, caecum; Co, colon; Sp, spleen; and Sm, skeletal muscle.

At 42 DPI, mice underwent a two-week immunosuppression protocol with cyclophosphamide to induce potential parasite reactivation in chronically infected tissues. One animal per group was randomly selected for bioimaging; following luciferin injection and euthanasia, organs were harvested and arranged as shown in **Figure 3A**, following the protocol described by (Costa et al., 2018). Preliminary analysis revealed varying numbers of parasite-positive organs across groups (**Figure 3B**). As expected, reactivation was observed in nearly all organs of untreated mice, whereas a minimal bioluminescent signal—limited to the stomach—was detected in mice treated with 100 mg/kg BNZ. Interestingly, radiance was quite low in organs derived from the BNZ 10/DFMO treated mouse in contrast to BNZ 10 mouse which displayed high bioluminescence intensity in gut mesenteries and colon besides other organs. Stomach, small intestine and caecum exhibited reactivation of the infection in the combined treatment whereas the liver and colon displayed very low signals (**Figures 3A, B**).

In summary, these data showed that low BNZ doses in combination with DFMO displayed a better anti-*T. cruzi* action than BNZ 10 individual therapy and confirm the major susceptibility of parasites to the combined treatment observed *in vitro*. It is also important to note that during the treatment period, the BNZ 100 group was the only one to exhibit side effects, which started 3 days post-treatment and persisted until the end of the study. These adverse effects included weight loss (averaging 5–7 g per mouse), light hypersensitivity, reduced mobility, and loss of muscle mass, and self-defense behaviors. Notably, the presence of DFMO in the BNZ 10/DFMO group did not lead to any clinical deterioration or worsening of symptoms compared to the group receiving BNZ 10.

## Discussion

Despite its discovery over 50 years ago, BNZ is still the gold standard for treating Chagas disease (Chagas disease, WHO, 2021). Its trypanocidal activity justifies its continued use, notwithstanding its high toxicity. It has been shown that treatment with BNZ can cure almost 100% cases of acute Chagas and, in some cases, can also prevent the disease progression and its most serious complications in the chronic infection stage. Benznidazole is activated by a parasite-specific nitroreductase to produce highly toxic reactive metabolites that damage *T. cruzi* by causing extensive DNA breaks, chromatin unpacking, and damaging proteins and lipids, ultimately leading to parasite death.

The major concerns against BNZ indication are the long duration of treatment (up to 2 months), and the presence of adverse reactions, which tend to be more frequent and more severe with increasing age. Side effects include allergic dermatitis, peripheral neuropathy, anorexia and weight loss, and insomnia, between others (Clinical Care of Chagas Disease, CDC). In the search for more effective and safer treatments, several strategies are currently being explored, including: (1) drugs targeting alternative *T. cruzi* pathways (Talevi et al., 2019; Beltran-Hortelano et al., 2022); (2) drugs with mechanisms of action similar to BNZ and NFX, such as fexinidazole (Imran et al., 2022); (3) novel drug delivery systems for BNZ (Li et al., 2021); and (4) combination therapies using low-dose BNZ to reduce toxicity and enhance tolerability while maintaining efficacy (Pecoul et al., 2016; Assmus et al., 2025). This work focuses on the latter approach as a proof of concept to increase BNZ susceptibility by impairing essential parasite nutrients required for antioxidant metabolism, cell division, and growth.

Based on the essentiality of PAs for *T. cruzi* intracellular development and the necessary acquisition of host cell spermidine for trypanothione synthesis, we firstly conducted infection experiments *in vitro* with the use of BNZ combined with DFMO, an irreversible inhibitor of host ODC. DFMO is a repurposed drug initially developed against cancer and currently recommended as a second line treatment against Human African trypanosomiasis (HAT) caused by *T. brucei* subspecies gambiense (Barrett, 2025), and in topical preparations to reduce hair growth in the facial hirsutism. DFMO was recently approved by FDA in the treatment of High-Risk Neuroblastoma (Kaneko et al., 2023) demonstrating the high versatility of this drug.

The effectiveness obtained for the BNZ/DFMO combination on amastigote growth in cell culture can be explained by the increased susceptibility of parasites to the prooxidant effect of BNZ in the presence of DFMO. As demonstrated previously, DFMO reduces the spermidine levels in the host cell (Vanrell et al., 2013b), decreasing the Spd availability for the parasites and subsequently impairing trypanothione synthesis. We do not discard other possible trypanocidal actions by the absence of Spd and the other PAs. Dr. Pereira´s laboratory repurposed isotretinoin to block PAs transport in *T. cruzi* and demonstrated that it causes nutrient starvation that triggers autophagic and apoptotic processes (Reigada et al., 2017).

*In vivo* studies further demonstrated the synergistic effect of DFMO when combined with low-dose BNZ. Using bioluminescent parasites to monitor the dynamic distribution of infection (Taylor et al., 2021), we observed that this combination significantly improved therapeutic outcomes by reducing both parasitemia peak and total parasite burden overtime. Notably, statistical analysis showed that the infection progression in mice receiving the combination treatment was comparable to those treated with a full dose (100 mg/kg/day) of BNZ.

In agreement with previously published data with the bioluminescent *T. cruzi* CL Brener strain (Lewis et al., 2015), chronic infection with Tul-Luc-mNeonGreen in BALB/c mice is characterized by the persistence of parasites in the gastrointestinal tract and other tissues and organs, skeletal muscle, lung, kidney, liver and spleen but not in the heart. The combined treatment resulted in a minor reactivation of infection after mice immunosuppression in the chronic stage. While BNZ 10 treated animals displayed the presence of parasites in 6 locations (gut mesentery, liver, colon, spleen, stomach and caecum) after immunosuppression, BNZ 10/DFMO-treated mice had parasites mainly in the stomach, with very low signals in liver, caecum, small intestine and colon. As expected, animals treated with BNZ 100 only displayed a minimal signal reflecting parasite persistence in the stomach, although this treatment significantly compromised the overall health of the mice. The comparable infection progression observed in both control and DFMO-treated mice, coupled with the lack of host cell toxicity *in vitro*, strongly suggests that the therapeutic benefit is driven by the sensitization of *T. cruzi* to BNZ via polyamine depletion, rather than an indirect effect on the host. In summary, this study provides new insights into the repurposing of DFMO as an adjuvant agent that boosts the efficacy of Benznidazole. The combined use of these drugs represents a proof of concept for the development of new therapeutic strategies to improve the treatment of Chagas disease, potentially increasing efficacy while reducing BZN-associated toxicity.

## Data Availability

The original contributions presented in the study are included in the article/**Supplementary Material**. Further inquiries can be directed to the corresponding author.

## Supplementary material

Supplementary Figure 1 Analysis of cell viability following BNZ and DFMO combination treatment. H9c2 cells were incubated with BNZ (0-250uM) and DFMO (0–5 mM) in a dose-combination matrix for 48 h. Cytotoxicity was assessed using the Alamar Blue (resazurin) assay; fluorescence was measured 6 h after reagent addition. The heat map illustrates the percentage decrease in cell viability according to the gray scale. Data represent the means of four independent experiments performed in duplicate.
